# The climate impact of high seas shipping

**DOI:** 10.1093/nsr/nwac279

**Published:** 2022-12-08

**Authors:** Yuze Li, Peng Jia, Shangrong Jiang, Haijiang Li, Haibo Kuang, Yongmiao Hong, Shouyang Wang, Xueting Zhao, Dabo Guan

**Affiliations:** Questrom School of Business, Boston University, Boston, MA 02215, USA; Collaborative Innovation Center for Transport Studies, Dalian Maritime University, Dalian 116026, China; School of Maritime Economics and Management, Dalian Maritime University, Dalian 116026, China; School of Economics and Management, University of Chinese Academy of Sciences, Beijing 100190, China; School of Economics and Management, University of Chinese Academy of Sciences, Beijing 100190, China; Collaborative Innovation Center for Transport Studies, Dalian Maritime University, Dalian 116026, China; School of Maritime Economics and Management, Dalian Maritime University, Dalian 116026, China; Collaborative Innovation Center for Transport Studies, Dalian Maritime University, Dalian 116026, China; School of Maritime Economics and Management, Dalian Maritime University, Dalian 116026, China; School of Economics and Management, University of Chinese Academy of Sciences, Beijing 100190, China; Academy of Mathematics and Systems Science, Chinese Academy of Sciences, Beijing 100190, China; Center for Forecasting Science, Chinese Academy of Sciences, Beijing 100190, China; School of Economics and Management, University of Chinese Academy of Sciences, Beijing 100190, China; Academy of Mathematics and Systems Science, Chinese Academy of Sciences, Beijing 100190, China; Center for Forecasting Science, Chinese Academy of Sciences, Beijing 100190, China; School of Entrepreneurship and Management, ShanghaiTech University, Shanghai 201210, China; Collaborative Innovation Center for Transport Studies, Dalian Maritime University, Dalian 116026, China; School of Maritime Economics and Management, Dalian Maritime University, Dalian 116026, China; Department of Earth System Science, Tsinghua University, Beijing 100080, China; School of International Development, University of East Anglia, Norwich NR4 7TJ, UK

**Keywords:** high seas, international shipping, GHG emission, policy evaluation, emission drivers

## Abstract

Strict carbon emission regulations are set with respect to countries’ territorial seas or shipping activities in exclusive economic zones to meet their climate change commitment under the Paris Agreement. However, no shipping policies on carbon mitigation are proposed for the world’s high seas regions, which results in carbon intensive shipping activities. In this paper, we propose a Geographic-based Emission Estimation Model (GEEM) to estimate shipping GHG emission patterns on high seas regions. The results indicate that annual emissions of carbon dioxide equivalent (CO_2_-e) in shipping on the high seas reached 211.60 million metric tonnes in 2019, accounting for about one-third of all shipping emissions globally and exceeding annual GHG emissions of countries such as Spain. The average emission from shipping activities on the high seas is growing at approximately 7.26% per year, which far surpasses the growth rate of global shipping emission at 2.23%. We propose implementation of policies on each high seas region with respect to the main emission driver identified from our results. Our policy evaluation results show that carbon mitigation policies could reduce emissons by 25.46 and 54.36 million tonnes CO_2_-e in the primary intervention stage and overall intervention stage, respectively, with 12.09% and 25.81% reduction rates in comparison to the 2019 annual GHG emissions in high seas shipping.

## Teaser

GHG emission on high seas could become a tragedy of the commons in the global maritime industry, which can be regulated by the corresponding carbon policies.

## INTRODUCTION

Over the past decades, accelerated international and regional maritime trading activities have boosted worldwide development of ocean-going shipping industries. The associated shipping greenhouse gas (GHG) emissions, however, have gradually become a non-neglectable issue against worldwide decarbonation and climate change goals [1]. As estimated by the International Maritime Organization (IMO) voyage-based methods of international shipping accounted for 755 million metric tonnes of annual GHG emissions in 2018 [2]. In order to improve emission reduction in the maritime industry and to meet climate change commitments under the Paris Agreement, many countries have submitted concrete plans and implemented strict carbon emission regulations for shipping activities in their territorial waters or exclusive economic zones (EEZs), such as emission-controlled areas, alternative fuel substitution, electric or nuclear propulsion adoption and renewable energy propulsion assistance, etc. [3–5] Previous maritime-related studies focused on territorial waters and EEZs carbon mitigation policy effectiveness evaluation and improvement [6,7]. While the Paris Agreement clearly outlines the emission reduction plans for each country's territorial waters, little attention has been paid to the fast-growing emissions on the international high seas. Due to the non-sovereign aspect of high seas, no signatories are directly responsible for high seas carbon emission reduction under the Paris Agreement and, thus, there have been no carbon mitigation policies or environmental regulations proposed or implemented in these regions. As a result, ships travelling on the high seas often operate in economically efficient manners, such as utilizing heavy fuel oil and travelling at high speeds, at the expense of environmental drawbacks [8,9]. Since the high seas account for more than two-thirds of the world’s ocean regions, carbon intensive shipping activities could become a potential barrier against worldwide carbon mitigation and sustainability efforts.

In comparison to the top-down approach utilized by previous maritime GHG inventory estimation studies, bottom-up approaches enable more accurate estimation results by summing up detailed individual ship emission outputs [10,11]. There are currently two bottom-up approaches used in the existing literature to calculate shipping GHG emissions for certain regions or countries, namely vessel-based and voyage-based. However, these methods rely heavily on two assumptions: the vessel-based method assumes that vessels of a similar type and age have uniform shipping behaviors; the voyage-based method assumes that international shipping emissions are those occurring on a voyage between two ports in different countries [2,12]. In this paper, we propose a new Geographic-based Emission Estimation Model (GEEM) to estimate international shipping GHG emission patterns on high seas regions. By incorporating the IHS Market Maritime & Trade vessel technical specification data and Automatic Identification System (AIS) data as our GEEM static and dynamic datasets, respectively, our GEEM method can be viewed as a bottom-up approach that identifies GHG inventories through broadly covered individual ship navigation information. In comparison to the existing two bottom-up GHG emission approaches, our GEEM method utilizes real-time data of geographic coordinates, which enables more accurate and robust high seas emission estimation by relaxing the aforementioned assumptions. In particular, we exclude international vessels that navigate between two countries’ EEZs from high seas GHG emissions, and we include domestic vessels whose routes cover high seas regions. In essence, our GEEM approach collects shipping navigation and emission data only for those occurring geographically on the high seas, and accordingly estimate our high seas shipping GHG emission results.

Our GEEM results indicate that the annual shipping carbon dioxide equivalent (CO_2_-e) emissions on the high seas reached 211.60 million metric tonnes per year (Mmt/yr) in 2019, accounting for about one-third of all global shipping emissions. The high seas shipping emissions in 2019 exceeds annual greenhouse gas emissions of countries such as Spain, Argentina and United Arab Emirates. More alarmingly, the average emission from shipping activities on the high seas is growing at approximately 7.26% per year, which far surpasses the global shipping emission growth rate of 2.23% per year [1]. By classifying the worldwide high seas into eight geographic regions and incorporating detailed vessel dynamic data of all ships from all routes, we find that there exists a great degree of heterogeneity in key factors that drive the shipping emission patterns across different high seas regions. As suggested by commonly-adopted maritime carbon mitigation regulations, we propose the primary implementation policies on each high seas region with respect to the main emission driver identified from our results. Our evaluation results show that carbon mitigation policies could reduce emissions by 25.46 and 54.36 million tonnes CO_2_-e in the primary intervention stage and overall intervention stage, respectively, with 12.09% and 25.81% reduction rates in comparison to the 2019 annual high seas shipping GHG emissions. Indeed, to regulate high seas shipping activities via the global maritime industry effort, international high seas shipping can contribute to world trading and economic growth in a more environmentally-friendly manner.

## RESULTS

### Shipping activities and emissions on the high seas

**Figure 1.**
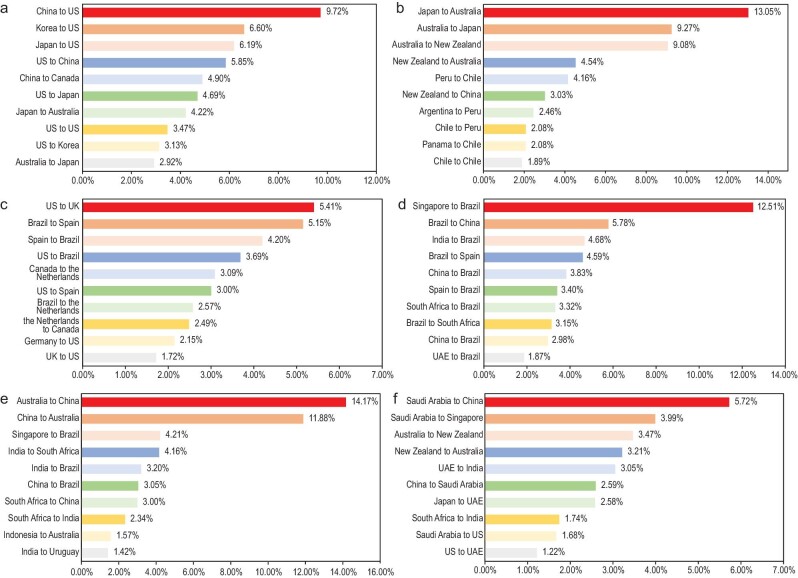
High seas shipping mileage distribution. Based on our GEEM calculation results, (a–f) report the average top 10 shipping mileage routes for North Pacific Ocean High Sea (a), South Pacific Ocean High Sea (b), North Atlantic Ocean High Sea (c), South Atlantic Ocean High Sea (d), Indian Ocean High Sea (e), and Other High Seas (f). The specific percentages in (a–f) indicate shipping mileage proportions of each high seas region during our GEEM sample period.

In this study, we follow the high seas division standard provided by the International Hydrographic Organization and classify the worldwide high seas into eight geographic regions, namely the North Pacific Ocean High Seas, South Pacific Ocean High Seas, North Atlantic Ocean High Seas, South Atlantic Ocean High Seas, Arctic Ocean High Seas, Southern Ocean High Seas, Indian Ocean High Seas, and Other High Seas. Using an AIS-based method, we first calculate the annual shipping activities of each region from 2015 to 2019 by adding up the distance travelled (in nautical miles) from all routes for all ships within the respective areas. Of the total shipping activities in 2019, about 23.43% were from the North Atlantic Ocean High Seas, which reflects the high traffic between European, North American, and South American countries. As shown in Fig. 1, the shipping activities between the United States and the United Kingdom, Brazil and Spain, as well as United States and Brazil are amongst the busiest routes in the region. The Indian Ocean High Seas accounted for approximately 20.98% of total shipping activities, which can be mainly attributed to the shipping activities among Asian countries. In particular, the shipping route between China and Australia is responsible for nearly 30% of the traffic in the region. The North Pacific Ocean High Seas is another region with significant shipping activities, contributing about 20.55% as most of the shipping routes between Asia and North America pass through this area. In particular, the shipping routes from China to the United States, Korea to the United States, and Japan to the United States exhibit the most amount of traffic in the region. Overall, the resulting total shipping mileage in the high seas has exceeded a total of 1.56 billion nautical miles (nm) in 2019 with an average annual growth rate of 6.81% in the past 5 years. The review of maritime transport by the United Nations Conference on Trade and Development (UNCTAD) indicates that international maritime trade expanded at 4.7%–6.7% annually from 2015 to 2019, with total volumes amounting to 11 billion tons in 2018 [13]. The accelerated international maritime trade volumes boost shipping activities on the high seas, especially for those rapidly developing shipping routes such as South–South trade, Belt and Road Initiative by China, Panama Canal– and Suez Canal–related seaborne trade [13–15].

We collect and process 79 613 vessels’ high seas shipping records in total, which represent a majority of the international shipping fleet. We also utilize the 3-minute frequency AIS data spanning from January 2015 to December 2019, which accounts for a total of 5.03 TB raw AIS dataset. Using the detailed AIS messages transmitted by all ships from all routes, we calculate CO_2_, SO_2_, NO_2_, particulate matter 2.5 (PM_2.5_), particulate matter 10 (PM_10_), CO, non-methane volatile organic compounds (NMVOCs), CH_4_ and N_2_O emissions generated by all vessels for each of the eight world high seas regions from 2015 to 2019. We calculate the CO_2_-e by switching other pollutants to CO_2_ to standardize the climate impact of high seas GHG emissions. The results indicate that the rapid development of shipping activities has resulted in a significant increase in emissions in the identified high seas regions. During this time, total CO_2_-e emissions rose from 163.98 Mmt/yr to 211.60 Mmt/yr, accounting for about one-third of all global shipping emissions. At the international level, the total high seas shipping-related CO_2_-e emissions in 2019 exceeds the total annual greenhouse gas emissions of countries such as Spain, Argentina and United Arab Emirates (countries’ emission data are available at www.globalcarbonatlas.org). That is to say, international shipping GHG emissions on the high seas could become an increasing barrier against worldwide carbon mitigation and sustainability efforts.

**Figure 2.**
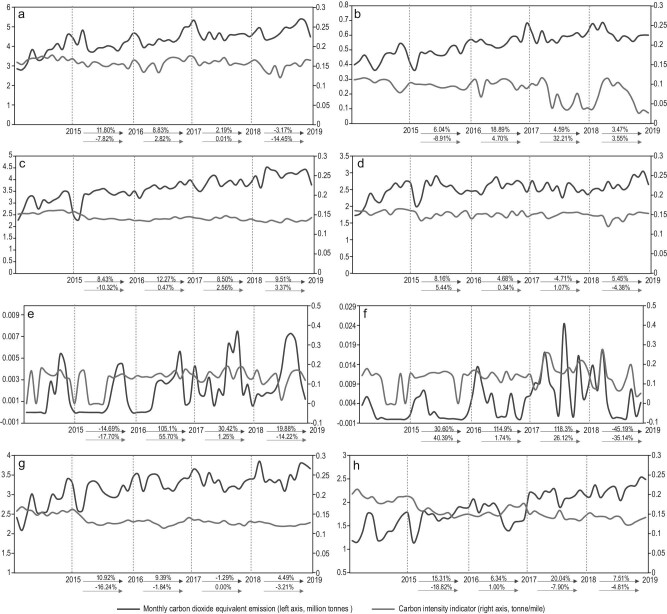
High seas shipping emission outputs and operational efficiencies. (a–h) provide the monthly carbon dioxide equivalent emissions and energy efficiency operational indicator for North Pacific Ocean High Seas (a), South Pacific Ocean High Seas (b), North Atlantic Ocean High Seas (c), South Atlantic Ocean High Seas (d), Arctic Ocean High Seas (e), Southern Ocean High Seas (f), Indian Ocean High Seas (g), and Other High Seas (h). The carbon intensity indicator (CII) is defined by the IMO as carbon dioxide emissions per actual cargo mile. The annual emission and efficiency growth rate are shown on the *x*-axis, with blue and red arrows, respectively.

Fig. 2 illustrates the changes in CO_2_-e emission output and carbon emission intensity across all high seas regions from 2015 to 2019. In terms of total emissions, the results reflect, to some degree, the intensity in shipping activities over the different high seas regions. Although the North Pacific Ocean High Seas ranks third in total mileage travelled, it is responsible for more than 25% of the total emissions generated. In fact, the North Pacific Ocean High Seas has the highest carbon emission intensity at 0.1481 tonne/nm, which suggests that the shipping activities of this area are more carbon intensive. The North Atlantic Ocean High Seas share is approximately 22%, while the Indian Ocean High Seas contributes about 18%. In terms of the average emission growth rate, the top three fastest growing emitting regions are the Other High Seas (14.56%), the North Atlantic Ocean High Seas (9.90%), and the South Pacific Ocean High Seas (9.11%). In particular, the North Atlantic Ocean High Seas ranks in the top three regions for both total emissions generated and emission growth rate, which indicates that the emission problem in one of the most heavily polluted regions has been increasing in severity. Although the South Atlantic High Seas only contributes 13% to the total emissions, emission intensity there is among the highest, reaching 0.1450 tonne/nm for CO_2_ emissions. Indeed, Fig. 2 shows the different GHG emission patterns of each high seas shipping activity in terms of total emission, emission growth rate and intensity. We provide more in-depth investigation on emission drivers of high seas shipping in the next section.

### Emission drivers on the high seas

**Figure 3.**
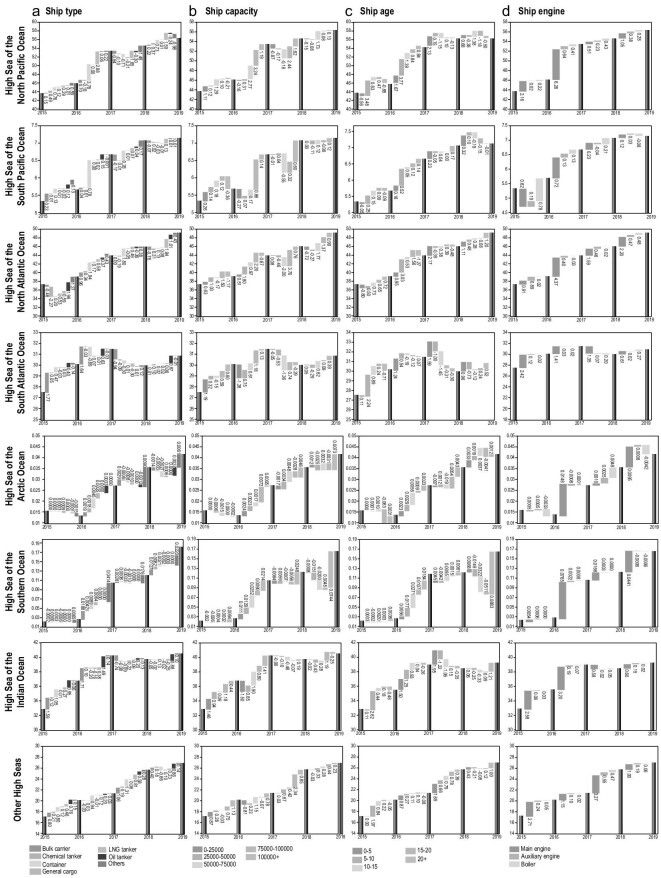
High seas shipping carbon emission drivers. The carbon dioxide equivalent emission compositions and structural changes of each high seas region are identified and classified by ship type (column a), ship capacity (column b), ship age (column c) and ship engine (column d). The dark blue bars indicate the annual individual high seas shipping emissions of carbon dioxide equivalent (million tonnes). The bars in bright colors (labeled in the legend) represent the annual emission contribution of the associated unit categories, which are noted with their respective emission outputs.

By incorporating detailed vessel and dynamic data of all ships from all routes, we find that there exists a great degree of heterogeneity in key factors that drive the shipping emission patterns across different high seas regions. As shown in Fig. 3, the degree of heterogeneity is mainly driven by the differences in key characteristics of ships active on each high seas region, namely, the ship type, capacity, age and engine usage. Since the emissions in the Arctic and Southern Ocean High Seas are nearly negligible (accounting for 0.02% and 0.08% of total high seas GHG emissions in 2019, respectively), we disregard them in the emission driver analysis. Therefore, results are shown for the six emission-significant high seas regions.

In terms of ship type, although there are 19 official types of vessel defined by the IMO, the top six most dominant ship types were responsible for nearly 80% of the total emissions while the other 13 types combined together for the rest. Thus, for the ease of analysis, we classify each vessel into one of seven major types based on its usage, namely bulk carrier, chemical tanker, container, general cargo, liquefied gas tanker (LG tanker), oil tanker, and others. According to the Energy Efficiency Operational Indicator (EEOI) defined in the IMO study, general cargo, liquefied gas tanker and container are the top three most carbon-intense ship types [2,16]. Fig. 3a illustrates the distribution of CO_2_ equivalent emissions across different ship types in each high seas region. We find that the emission patterns of the North Pacific Ocean High Seas and North Atlantic Ocean High Seas are alarmingly different from those of the other high seas regions. In particular, the top three carbon intense ship types were responsible for 62% and 54% of the emissions in the aforementioned two regions, respectively, while they were only responsible for 19%–25% in the rest. The high proportion of emissions from the carbon intense ships can be mainly attributed to the rapid growth in containers and liquefied gas tankers in these regions. Specifically, in the North Pacific Ocean High Seas, the emission contribution from liquefied gas tankers spiked from 2% to 13% between 2015 and 2019, resulting in a 175% growth in its amount of emissions in the region. Moreover, the annual emission growth rate for containers in the North Atlantic Ocean High Seas is 25%, which is much higher than the 5% average in the other regions. In contrast, we find that in regions such as the South Atlantic Ocean High Seas and Indian Ocean High Seas, low carbon intense ships such as bulk carriers and oil tankers are the dominant source of emissions.

In terms of ship capacity, following the guidelines from IMO ship capacity statistics and category [2], we classify all vessels into five dead-weight tonnage (dwt) groups, namely, 0–25 000 dwt, 25 000–50 000 dwt, 50 000–75 000 dwt, 75 000–100 000 dwt, and 100 000+ dwt. It is important to note that according to the EEOI defined in both the IMO and previous studies, ships under 50 000 dwt typically exhibit a higher carbon intensity than other ships [17]. Fig. 3b illustrates the distribution of CO_2_ equivalent emissions across different ship capacities. Out of all the high seas regions, the emission pattern in the South Atlantic High Seas stands out from the rest. In particular, the carbon-intensive ships under 50 000 dwt are responsible for over 21% of the total emissions in the area. In fact, the relatively lower dwt shipping pattern is consistent with the short shipping routes and varied goods demand for berthing ports in the South Atlantic High Seas. In contrast, the proportion of emissions attributed to ships of the same weight classes is only 10% in regions such as the North Pacific Atlantic High Seas.

In terms of ship age, following the guidelines from IMO classification convention, we classify all the vessels into five age classes: 0–5 years, 5–10 years, 10–15 years, 15–20 years, and 20+ years. It is a fact that most new-build ships install engines with a better EEDI and specific fuel consumption than ships with an older construction year. As illustrated in Fig. 3c, there are two distinct emission pattern distributions across all high seas regions. On the one hand, the North Pacific, South Atlantic, Indian and the Other High Seas have relatively newer ships as the dominant emission source. In particular, ships with a service age of less than 10 years contribute 42% of emissions for the North Pacific Ocean High Seas, 48% South Atlantic Ocean High Seas, 43% for the Indian Ocean High Seas, and 42% for the Other High Seas. Moreover, the oldest ships (20+ years) only account for an average of 10.71% of emissions in these regions over the five-year period. On the other hand, emission sources in the South Pacific Ocean High Seas primarily consist of older ships with service age greater than 15 years. In particular, the ships with service age over 20 years contribute 26% of emissions for the South Pacific, which is much higher than the average contribution rate in the other four high seas regions. In fact, in comparison to the top three carbon emission high seas regions, shipping emission regulations on territorial water and EEZ for countries along the shipping routes in the South Pacific Ocean are relatively loose due to less significant emission output. From a policy regulation perspective, shipping companies would allocate their older ships to the South Pacific Ocean and new-built ships on other Oceans, which results in ships with service age over 15 years as the major emission sources in the South Pacific High Seas.

In terms of ship engine usage, the majority of CO_2_ equivalent emissions are associated with main engines [18]. However, the Other High Seas stand out from the rest of the regions as the growth rate in main engine emission contribution reaches 10.2% annually, which is more than twice the average growth rate in emission contribution in other regions. Moreover, the auxiliary engine emission contribution in the Other High Seas is growing at 12.89% annually, far exceeding the 4.94% annual growth rate in other regions. This reflects the fact that the cruise distances in the Other High Seas region are much shorter than the rest of the high seas regions. Thus, the growth in emission share attributable to auxiliary engines is much higher.

### Shipping carbon mitigation policies evaluation

The Fourth IMO GHG Emission Study and previous works evaluate territorial water or EEZ shipping GHG emission mitigation policies’ effectiveness with respect to the mid-term (2030) and long-term (2050) reduction targets [19,20]. Following the current mid- and long-term policy settings on territorial water or EEZ, we examine the high seas GHG reduction effectiveness at both primary policy implementation stage and overall implementation stage. We propose that primary carbon mitigation policies be implemented on each high seas region based on its main emission driver identified in our results (see Table 1). Specifically, in the ship type (ST) policy, we target the top three carbon-intense ship types (general cargo, liquefied gas tankers, and containers) by substituting the heavy fuel oil (2.43% sulfur content) used in these ships with alternative fuel such as marine diesel oil and marine gas oil (0.13% sulfur content) [21]. As our findings in Fig. 3 suggest, the carbon emission contribution and emission growth rate of the carbon-intense ships in the North Pacific Ocean High Seas and North Atlantic Ocean High Seas far exceed those in other high seas regions. Thus, we directly target this primary emission driver by implementing the ST policy in these two regions; in terms of ship capacity, contrary to common perception, lower capacity ships typically have higher power output of the ships’ prime movers, which results in less fuel efficiency, higher fuel consumption rate, and higher unit emission output. In order to reduce the exhaust gas emissions, as findings from previous literature suggest, increased capacity of containerized shipping will decrease the amount of required fuel for the transported container, which ultimately reduces the emitted greenhouse gases for international shipping. In order to reduce the exhaust gas emissions from vessels, many navigational corporations have started to consider using mega or medium vessels [22]. Thus, for the ship capacity (SC) policy, we aim to improve the average international shipping capacity by shifting the shipping activities conducted through ships with capacities lower than 50 000 dwt to other ships. Since the proportion of emissions attributed to ships under 50 000 dwt in the Indian Ocean High Seas and South Atlantic High Seas are more than twice as much as that of other high seas regions, we directly implement the SC policy in these two regions. In terms of ship age, since newer vessels are typically equipped with better technologies and energy saving devices, they have higher carbon efficiency and lower emissions compared to older ships. Thus, for the ship age (SA) policy, we raise the in-service ship standard by implementing compulsory scrapping of active ships with service age greater than 20 years and substitute them with newly-built ships for high seas shipping activities [23]. Since the emission contribution rate of ships with service age over 20 years in the South Pacific far exceeds that in other high seas regions, we directly implement the SA policy in this region; for the ship engine (SE) policy, following guidelines from IMO to use ‘speed reduction as a measure to improve operational emission efficiency of existing ships’, we set speed reduction at 10% for all ship types on high seas shipping for main engine carbon emission mitigation [24]. Since the emission growth rate of main engines used in the Other High Seas is significantly higher than that in other regions, we directly implement the SE policy in this region.

**Table 1.** High seas shipping carbon mitigation policies and implementation strategies.

Ship categories	Intended carbon mitigation policies	Measures	Primary high seas area
Ship type	High emission ship type supervision	Alternative fuel adoption for general cargo, liquefied gas tankers and container	North Pacific Ocean High Seas; North Atlantic Ocean High Seas
Ship capacity	Shipping capacity intervention	Improve the average international shipping capacity	Indian Ocean High Seas; South Atlantic Ocean High Seas
Ship age	New-build ships substitution	Raise the in-service ship standard; Compulsory scrapping policy for old ships	South Pacific Ocean High Seas
Ship engine	Main engine improvements	High seas shipping speed reduction	Other High Seas

Note: Extensive carbon mitigation policies in shipping proposed by the International Maritime Organization and previous works. We collect the specific policies that target and foster maritime industry decarbonization through ship type, capacity, age and engine categories. We define the primary implementation policy with respect to the main shipping carbon emission driver of each high seas derived from our results. As a result, we evaluate the high seas carbon mitigation policy effectiveness in both primary implementation stage and overall implementation stage.

**Figure 4.**
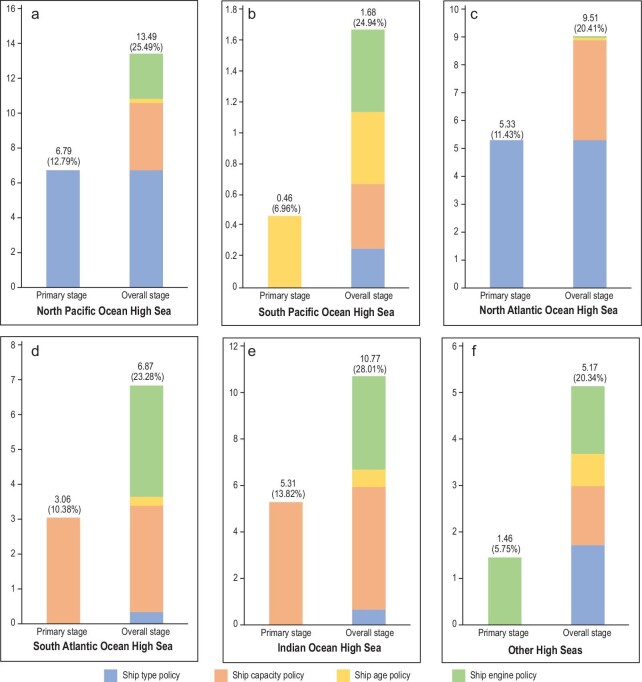
Evaluation of effectiveness of emission mitigation policies in High Seas Shipping. (a–f) provide the estimated carbon mitigation effectiveness of North Pacific Ocean High Seas (a), South Pacific Ocean High Seas (b), North Atlantic Ocean High Seas (c), South Atlantic Ocean High Seas (d), Indian Ocean High Seas (e) and Other High Seas (f). The primary and overall stages indicate the primary and overall policy implementation indicated in Table 1. The carbon reduction amount (million tonnes) and percentage of each high seas region are presented above each bar, respectively.

The policy effectiveness assessment is conducted through two stages. In the primary stage, each carbon mitigation policy is implemented separately in the target high seas region. After 2030, we enter the overall stage, where the carbon mitigation policies are implemented together across all the high seas regions. As illustrated in Fig. 4, our evaluation results indicate that implementing tailored carbon mitigation policies in different high seas regions could reduce 25.46 and 54.36 million tonnes of CO_2_ equivalent emission in the primary intervention stage and the overall intervention stage, respectively, with 12.09% and 25.81% reduction rates in comparison to the annual high seas shipping GHG emissions for 2019. In particular, it is worth noting that the regions with the greatest emission reduction rate in the primary stage are the regions with the greatest high seas shipping emission contribution rate overall, namely the Indian Ocean High Seas (13.82%), North Pacific Ocean High Seas (12.79%), and North Atlantic Ocean High Seas (11.43%). Moreover, the tailored carbon mitigation policy implemented through the primary stage shows the greatest emission reduction percentage in each of the high seas regions (an average 46.84% of the total emission reduction), which indicates that it is the most effective policy in reducing emissions in the particular region compared to other policies. Thus, by identifying the key factors driving the emission patterns in different high seas regions and accordingly designing tailored carbon mitigation policies for each region, it allows international high seas shipping to contribute to world trading and economic growth in a more environmentally-friendly manner.

## DISCUSSION

Climate change is a global issue that requires solutions based on international cooperation. To alleviate the negative impact of climate change, the Paris Agreement was signed by world leaders in 2015 to foster global greenhouse gas emission reduction across national borders [25–27]. Signatories are committed to reduce GHG emission from all industries and human activities, and all countries’ climate efforts are monitored and reviewed by the United Nations every 5 years [28]. In terms of the maritime industry, strict carbon emission regulations are set with respect to shipping activities in countries’ territorial water or exclusive economic zone (EEZ), such as emission-controlled areas, alternative fuel substitution, electric or nuclear propulsion adoption and renewable energy propulsion assistance, etc. [29,30]. For example, emission-controlled areas are proposed to limit SO_2_, NO_2_ and particulate matter emissions in major countries’ territorial sea shipping [31]; ships are required to use fuels of low sulfur content such as marine diesel oil and marine gas oil to reduce carbon emissions when the shipping activities occur at berth or at EEZs of US and East Asia areas [32,33]. As a result, the Fourth IMO GHG Emission Study suggests that the annual international shipping GHG emission growth gradually slowed down at a 2.23% average annual rate from 2015 to 2018 due to the associated maritime and international shipping carbon mitigation policy interventions.

However, current carbon mitigation policies in shipping are effective and implemented only in territorial seas and EEZs as part of a certain country's carbon reduction policies. Due to the non-sovereign aspect of high seas regions, no shipping carbon mitigation policies or environmental regulations are proposed or implemented for the world's high seas regions, as no signatories are responsible for high seas carbon reduction under the Paris Agreement [34,35]. For a lower operational cost of international shipping, ships usually navigate in an economically efficient manner on the high seas by utilizing heavy fuel oil and travelling with high speed without environmental concern. As a result, our GEEM estimation results indicate that the carbon intensive shipping activities have resulted in a significant increase in emissions on high seas regions. The total CO_2_-e emissions reached 211.60 Mmt/yr in 2019, which exceeds the total annual greenhouse gas emissions of countries such as Spain, Argentina, and United Arab Emirates. In addition, the average emission from shipping activities on the high seas is growing at approximately 7.26% per year, which far surpasses the global shipping emission growth rate of 2.23% per year. In essence, without policy intervention, international shipping GHG emissions on the high seas could become a tragedy of the commons in the global maritime industry: individual ships behave on their own interests to maximize their high seas shipping profits, ignoring the negative externality and climate change impact of their carbon intensive shipping patterns.

Utilizing our GEEM bottom-up vessel dynamic statistics of all ships from all routes, we find heterogeneity in key factors that drive the shipping emission pattern across different high seas regions in terms of ship type, capacity, age and engine categories. In order to evaluate the effectiveness of different carbon mitigation policies on high seas shipping, we collect the specific policies that target fostering of maritime industry decarbonization in territory seas and EEZs and propose the primary implementation policy with respect to the main emission driver of each high seas region identified from our results. Specifically speaking, we set and evaluate ship type policies on the Other High Seas, ship capacity policy on the Indian and South Atlantic High Seas, ship age policy on the South Pacific High Seas, ship engine policy on the North Pacific and North Atlantic High Seas.

Although our evaluation results indicate that the carbon mitigation polices can effectively reduce shipping GHG emissions on the high seas, implementing these policies may be difficult as no signatories are directly responsible for these regions. To facilitate international cooperation and the development of targeted regional high seas emission control agreement between countries, we also identify the major emission-contributing shipping routes and the key signatories involved in each high seas region as shown in Fig. 1. Specifically, the trading activities between China, the United States, Korea, Japan and Canada contribute to nearly 50% of the emissions generated in the North Pacific Ocean High Seas. In the South Pacific Ocean High Seas, the shipping routes between Japan, Australia and New Zealand are the most carbon emission intensive, generating 36% of the emissions in the region. In the North Atlantic Ocean High Seas, the trading routes with the heaviest traffic and carbon emissions involve countries such as the United States, the United Kingdom, Brazil, Spain, Canada, the Netherlands and Germany. In the South Atlantic Ocean High Seas, the top three most carbon-intensive trading routes are from Singapore to Brazil, Brazil to China, and India to Brazil, contributing 13%, 6%, and 5%, respectively. In the Indian Ocean High Seas, the shipping routes between China and Australia are especially carbon intensive, contributing to over 35% of the emissions in the region. In the Other High Seas Region, some of the most carbon intensive trading routes involve countries such as China, Saudi Arabia, Singapore, Australia, New Zealand, United Arab Emirates and South Africa. By identifying these emission-intensive trading routes, it can encourage the key countries involved to form regional high seas emission control agreements and impose the carbon mitigation policies discussed above on the vessels operating along these trading routes. After all, by regulating high seas shipping activity via the international cooperation and global maritime industry effort, international high seas shipping can contribute to world trading and economic growth in a more environment-friendly manner in the future.

## METHODS

### GEEM static database

In this study, a new Geography-based Emission Estimation Model (GEEM) static database was created using the vessel technical specification dataset provided by the IHS Market Maritime & Trade and AIS static database. The IHS and AIS static database contains ship characteristics for vessels as of 2020. The ships range from 100 GT fishing ferries and service vessels to the largest bulk carriers and cargo ships, covering both ships that engage in international as well as domestic navigation. In this study, the combined IHS and AIS static database contains all the data collected and updated to 2020. Thus, we checked each vessel's status against a timestamp of the most recent change in status separately to ensure that only ‘in service’ vessels are included in our GEEM static database.

The GEEM static database provides detailed ship characteristics including the IMO number, vessel type, build year, length, width, height, capacity, designed speed, fuel type, installed engine power, engine RPM, maximum draught, dead weight tonnage (dwt), etc. This wide range of metrics is essential for estimating fuel consumption and emission from ships. However, the data is sometimes incomplete as one or more pieces of technical information were found to be missing for some ships in the IHS dataset. In our data, 0.06% of the ships are missing capacity, 2.4% of the ships are missing build year, 3.0% of the ships are missing fuel type, 20.7% of the ships are missing design speed, and 26.4% of the ships are missing engine RPM. Simply excluding those particular ships with missing technical information from our calculation or assigning default values to the missing property will lead to significant computational inaccuracies. To correct the data and address the uncertainty, we designed a robust method to infill these missing technical specifications. Following the guidelines recommended by the Fourth IMO GHG Study [2]. We create a multilinear regression for each ship type by taking into account individual vessel's known design parameters such as beam, draught and capacity. Since both beam and draught serve as the basis in the estimation of other metrics, the missing values for these metrics are first filled based on median values per type and size category. After this essential information is infilled, we apply individual regressions on each of the other metrics. Finally, for individual ships that could not be infilled due to too many missing entries, we replace the missing information with the median values of their respective type and size class.

In this analysis, the technical specification data were collected and pre-processed for 79 613 vessels, which represents a majority of the international shipping fleet. The fleet scale is reasonable compared with the previous literature [36,37]. The original vessels collected are categorized into 19 vessel type categories according to the IMO ship types classified in the Fourth IMO GHG Study. In addition, the vessels collected are also classified into four age groups based on their build years. Table S1 lists the classified vessel types, and the number of vessels counted in each category. Table S2 lists the classified engine tier groups, vessel capacity group and the number of vessels counted in each group.

### Dynamic ship movement database

One of the advantages of this study is the superior quality Automatic Identification System (AIS) data in the high seas regions across the globe. In 2002, the AIS was introduced by the IMO International Convention for the Safety of Life at Sea (SOLAS) to improve maritime safety. Acting as a dynamic tracking and monitoring system, the AIS provides broad coverage and delivers detailed real-time information on the ship, including a ship's identity, position, speed, and draught at a given timestamp. Following the mandate set by IMO SOLAS, all ships over 300 GT engaged in international voyages, cargo ships over 500 GT engaged in national voyages and all passenger ships are required to install an AIS transceiver. According to the most recent study [38–40], the number of ships equipped with AIS and the number of AIS messages transmitted per year has experienced significant growth, which suggests that the introduction of the automatic vessel position reporting system has significantly reduced the uncertainty concerning ship activities and their geographical distribution.

This study utilizes the AIS dataset to construct our GEEM dynamic database. That is to say, our GEEM dynamic database includes metrics that are essential for the analysis of vessel movement and activity, such as the IMO identification number, Maritime Mobile Service Identify (MMSI) code, vessel coordinate (longitude and latitude), vessel actual speed, voyage draught, and time information. As shown in Table S3, the AIS transmission rate is consistent with the vessel's moving status and transponder type. All the AIS data are transmitted with a broadcast frequency of one message for no more than 3 minutes. As a result, the study utilizes the full year 3-minute frequency AIS data spanning from January 2015 to December 2019, which accounts for a total of 5.03 TB raw AIS dataset.

### Research domain identification

As suggested by the United Nations Convention on the Law of the Sea, high seas can be defined as all parts of the sea that are not included in the exclusive economic zone (EEZ). In this study, we first identify the worldwide high seas by subtracting the country's EEZs from the world sea boundary. In addition, following the high seas division standard provided by the International Hydrographic Organization, we divide the worldwide high seas into eight geographic regions and define each high seas region's longitude and latitude based on the geographic information system (GIS) database (namely the North Pacific Ocean High Seas, South Pacific Ocean High Seas, North Atlantic Ocean High Seas, South Atlantic Ocean High Seas, Arctic Ocean High Seas, Southern Ocean High Seas, Indian Ocean High Seas, and Other High Seas). The GHG emissions of vessels are collected and aggregated to respective high seas region by 0.05° grid box based on their AIS messages. By dividing the worldwide high seas into eight geographic regions, we are able to investigate the different GHG emission patterns and emission drivers of each high seas region, and the policies can be designed and evaluated in a more specific and effective manner.

Previous studies use bottom-up vessel-based or voyage-based methods to calculate shipping GHG emissions for certain regions or countries [2,41,42]. In essence, these methods rely heavily on their assumptions: the vessel-based method assumes that vessels with similar type and age have uniform shipping behaviors; the voyage-based method distinguishes international and domestic shipping emissions as those which occurred on a voyage between two ports in different or same countries.

In this paper, we propose a new geographic-based method for estimating the high seas shipping GHG inventory. As illustrated in Figure S1, no matter the vessel type, age or the shipping destination, AIS messages are collected only when shipping occurred geographically on the high seas. Based on the bottom-up approach, this paper uses the spatial join method in GIS spatial superposition analysis to identify ship trajectory points located in different high seas regions. We then obtain high seas emissions by accumulating ship trajectory emissions in each high seas region. In comparison to the existing two bottom-up GHG emission approaches, the geographic-based method enables more accurate and robust high seas emission estimation due to the following aspects: international vessels navigate between two countries’ EEZs are excluded from high seas GHG inventory (such as Port A Country A to Port A Country B in Figure S1); domestic shipping would be accounted as high seas GHG emission if the shipping route covers a high seas region (such as Port A Country A to Port C Country A in Figure S1).

### Geographic-based Emission Estimation Model

The technical strategy of our proposed Geographic-based Emission Estimation Model (GEEM) is illustrated in Figure S2. As discussed above, we utilize the IHS raw database and vessel AIS messages to construct our GEEM static and dynamic database. We design a geographic-based AIS messages collection method to classify and categorize high seas shipping route and associated GHG emission. In this section, we demonstrate the detailed GEEM high seas emission calculation method, the specifications of emission equation settings and the updated emission factors used in this study.

In international shipping, the GHG emission of each vessel is produced by three types of vessel engines, namely the main engine (propulsion engine), the auxiliary engine and the boiler. Since the main engine and the auxiliary engine are the moving power source of shipping, the emission intensities of the main and auxiliary engine are determined by a variety of shipping characteristics such as the vessel movement modes, instantaneous load factors, and maximum continuous rated power, etc. The boiler is used for hot water production and fuel heating. Its emission intensity is mainly associated with vessel fuel types. We next show how our GEEM approach calculate and collect the GHG emissions of the above three engines and formulate the total emission of high seas from a bottom-up approach.

The GHG emission of each vessel *i* can be calculated as the sum of vessel main engine, auxiliary engine and boiler emission:

(1) \begin{equation*}E\, = \,{E}_{\textit {main}}\, + \,{E}_{\textit {auxiliary}}\, + \,{E}_{\textit {boiler}}.\end{equation*}

In this study, we formulate the total GHG emission *TE* of each high seas region by summing up the individual vessel's emission occurring in high seas regions geographically:

(2) \begin{equation*}{\textit {TE}}_i\, = \,\sum\limits_{i = 1}^n {{E}_{i,j}}. \end{equation*}

In terms of the vessel main engine, the GHG emission of the engine can be expressed as follows:

(3) \begin{eqnarray*} {E}_{\textit {main}} &=& {\textit {MCR}}\, \times \,E{F}_{\textit {main}}\, \times \,\sum\limits_{j\, = \,1}^m L{F}_j \\ && \times \,A{F}_j\, \times \,\Delta {T}_j , \end{eqnarray*}

where *MCR* is the maximum continuous rated power; $E{F}_{\textit {main}}\,$ represents the emission factor of vessel main engine; ${\textit {LF}}_j$ is the instantaneous load factor at time *j*, $A{F}_j$ is the emission adjustment factor when the vessel's instantaneous load factor is lower than 20%. $\,\Delta {T}_j$ is the time span of the two adjacent AIS messages. It is worth noting that the emission factor for vessel main engine and auxiliary engine provided by the previous studies lack the High Speed Diesel (HSD) and Slow Speed Diesel (SSD) emission factors, respectively. In this study, we update the overall vessel main and auxiliary engine emission factors based on the IMO Fourth GHG Emission Study. In addition, we also calculate the Tier 3 (vessel construction date after 2016) vessel's GHG emission intensities for both the main and auxiliary engine. The emission factors for the main engine are reported in Table S4.

The instantaneous load factor $L{F}_j$ in Equation (4) at time *j* can be formulated as follows:

(4) \begin{equation*}{\textit {LF}}_j\, = \,{\left( {\frac{{{v}_j}}{{\textit {MDS}}}} \right)}^3,\end{equation*}

where ${v}_j$ is the vessel's instantaneous speed at time *j* and *MDS* is the vessel's maximum designed speed. The base emission factors for vessel main engine would decrease by about 20% load. As a result, according to the adjusted emission factor statistics of Energy and Environmental Analysis, Table S5 shows the adjusted emission factors $A{F}_j$ for vessel main engines at low loads.

In terms of vessel auxiliary engine, the GHG emission of auxiliary engine can be expressed as follows:

(5) \begin{equation*}{E}_{\textit {auxiliary}}\, = \,E{F}_{\textit {auxiliary}}\, \times \,\sum\limits_{j = 1}^m {{P}_{au{x}_j}} \times \Delta {T}_j,\end{equation*}

Where $\,E{F}_{\textit {auxiliary}}$ is the emission factor of vessel auxiliary engine at certain fuel types, ${P}_{\_au{x}_j}$ is the auxiliary engine power output at time *j*, and the $\Delta {T}_j$ is the time span of the two adjacent AIS messages. Emission factors for auxiliary engine are reported in Table S6. In fact, the vessel auxiliary engine ${P}_{\_au{x}_j}$ and boiler have different power outputs when the vessel movement mode changes. Vessel movement modes can be categorized into four types by their speed and maximum continuous rated power (MCR), namely At berth (speed less than 1 knot), At anchorage (speed between 1 knot and 3 knots), Maneuvering (speed great than 3 knots and less than 20% MCR) and At sea (speed above 20% MCR). As a result, Table S7 provides adjusted auxiliary engine and boiler power outputs for different vessel movement modes.

GHG emission of vessel boiler can be expressed as follows:

(6) \begin{equation*}{E}_{\textit {boiler}}\, = \,E{F}_{\textit {boiler}}\, \times \,\sum\limits_{j\, = \,1}^m {{P}_{\textit {boile}{r}_j}} \, \times \,\Delta {T}_j,\end{equation*}

where $E{F}_{boiler}$ is the emission factor of vessel boiler at certain fuel types, ${P}_{boile{r}_j}$ is the boiler power output at time *j*, and the $\Delta {T}_j$ is the time span of the two adjacent AIS messages. In fact, the emission intensity of vessel boiler can generally be determined by sulfur content of vessel fuels. As suggested by the IMO Maritime Environment Protection Committee (www.imo.org), we identify three sulfur content categories by different fuel types. Table S8 summarizes the emission factor for vessel boiler in this study.

### Emissions uncertainty analysis

The uncertainty in our global high seas shipping emissions estimation mainly originates from the following sources: (1) the uncertainty of the static dataset; (2) the uncertainty of dynamic dataset in the sampling of AIS data; (3) the uncertainty of emission factors of the observed fleet ships.

The uncertainty of the static dataset mainly includes the ship specification parameters and ship coverage. In this study, the ship specifications are obtained from the official IHS database and improved using our adaptive infilling algorithm. As shown in Table S9, we compare our static dataset to the official IMO dataset in terms of numbers and coverage. Although we exclude ships that have no high seas shipping records, our GEEM static dataset collects 79 613 vessels for all types in sum, and the majority of 19 ship types covers 80% higher of the IMO dataset. In terms of the top six GHG emission contributors of ship type on high seas shipping, namely bulk carrier, chemical tanker, container, general cargo, liquefied gas tanker and oil tanker, our dataset covers 94.9% of the IMO dataset. That is to say, the GHG estimation results of our proposed GEEM method is built on a well-covered static dataset.

The data quality of the dynamic database is evaluated according to three aspects: the AIS data coverage rate, AIS time interval frequency, and redundant data. The first step in calculating the AIS coverage rate is to check the GEEM static database and determine all the vessels that are active during the given period of this study. To do so, a set of rules are applied based on each vessel's build year, the current ship status, and the year that the vessel's ship status was last updated. Next, all the active vessels are matched with the messages in the AIS database. Table S10 describes the size of the total fleet classified as in service with the percentage that also appears in the AIS database. For top six high seas emission ship types, 94.6% of the six ship types are observed with AIS.

Table S11 shows the time interval statistics for our AIS data. Among all the archived messages, 96.77% of our AIS data have a time interval of less than 2 minutes. Only about 0.05% of our AIS data have a time interval of more than 30 minutes, which may be caused by extreme weather situations, complicated geographical condition interferences and other uncertain reasons. For these long interval messages (two signals have intervals longer than 600 seconds), speeds between each two of the AIS signals were generated by interpolation for every 600 seconds along the voyage trajectory.

In addition to the successfully matched vessels, AIS messages received from other vessels could not be matched due to a lack of technical data. Although omitting emissions from these unmatched ships introduces uncertainty, the existing studies have shown that its impact on emission estimation is rather negligible [43,44]. Another concern for matching the AIS data with the GEEM static database is message duplication. Since AIS data are reported from multiple AIS transceivers, both terrestrial and satellite based, there exists a possibility that duplicated messages may be received and recorded. To eliminate the occurrence of this phenomenon, we first put all the AIS messages collected for each individual ship in a sequential manner. When each ship's AIS data are ranked according to time sequence, the duplicated messages will have a time interval of 0, and therefore can be easily discovered and discarded. Thus, the duplicated messages will not affect our final emission calculation result.

The uncertainties of emission factors include the main engine, the auxiliary engine and the boiler. As discussed in previous sections, we update the overall vessel main and auxiliary engine emission factors based on the official IMO Fourth GHG Emission Study. In addition, we also calculate the 2019 vessel's GHG emission intensities for all engine types. As reported by the Fourth IMO GHG emission study [2], the results from the Monte Carlo analysis for international shipping CO_2_-equivalent emissions indicate that the uncertainty in the average CO_2_-equivalent emission factor is relatively small and set as 2.68% standard deviation of mean. However, international fleets have different carbon emission factors as for some pollutants such as SO_2_, PM_2.5_ and PM_10_ during our sample period. As a result, we update the yearly emission factors for the above three pollutants based on the IMO Fourth GHG Emission Study. Tables S12 and S13 demonstrate the updated PM_10_ (the emission factors of PM_2.5_ are 92% of PM_10_) and SO_2_ emission factor for all engine and fuel types, respectively.

## DATA AVAILABILITY

The GEEM static and dynamic datasets generated and analyzed during the current study are available on Google Drive (https://drive.google.com/file/d/1JckaZrWeA1Ez9MR-6q1ODgT23KApJaKD/view?usp=sharing). All data are also available from the corresponding author upon reasonable request.

## CODE AVAILABILITY

The Geographic-based Emission Estimation Model codes are available on GitHub (https://github.com/liyu28/High_Seas_Shipping.git). All codes are also available from the corresponding author upon reasonable request.

## Supplementary Material

nwac279_Supplemental_File
