# Supplementary material for: The climate impact of high seas shipping
Source: Natl Sci Rev. 2022 Dec 8;10(3):nwac279. doi: 10.1093/nsr/nwac279 (PMC9976761; doi:10.1093/nsr/nwac279)
Supplement: nwac279_Supplemental_File [file nwac279_supplemental_file.docx]

**Supplementary Information for**

**The Climate Change of High Seas Shipping**

Yuze Li^1,#^, Peng Jia^2,3,4,#^, Shangrong Jiang^4,#^, Haijiang Li^2,3^, Haibo Kuang^2,3^, Yongmiao Hong ^4,5,6^, Shouyang Wang^4,5,6,7,^^[[1]](#footnote-1)^*, Xueting Zhao^2,3^, Dabo Guan^8,9,*^

^1^ Questrom School of Business, Boston University, Boston Massachusetts 02215, United States

^2^ Collaborative Innovation Center for Transport Studies, Dalian Maritime University, Dalian 116026, China

^3^ School of Maritime Economics and Management, Dalian Maritime University, Dalian 116026, China

^4^ School of Economics and Management, University of Chinese Academy of Sciences, Beijing 100190,China

^5^ Academy of Mathematics and Systems Science, Chinese Academy of Sciences, Beijing 100190, China

^6^ Center for Forecasting Science, Chinese Academy of Sciences, Beijing 100190, China

^7^ School of Entrepreneurship and Management, ShanghaiTech University, Shanghai, 201210, China

^8^ Department of Earth System Science, Tsinghua University, Beijing 100080, China

^9^ School of International Development, University of East Anglia, Norwich NR4 7TJ, UK

^#^ These authors are co-first authors and contributed equally: Yuze Li, Peng Jia, Shangrong Jiang

**Table S1| GEEM static dataset vessel types**

| Ship type | This study |
| --- | --- |
| Bulk Carrier | 11981 |
| Oil Tanker | 7403 |
| Container | 5366 |
| General Cargo | 13196 |
| Chemical Tanker | 5145 |
| Ro-Ro | 1162 |
| Cruise | 475 |
| Refrigerated Bulk | 684 |
| Liquefied Gas Tanker | 2011 |
| Other Liquids Tankers | 34 |
| Ferry - Pax Only | 1272 |
| Ferry - Ro-Pax | 2120 |
| Vehicle | 842 |
| Service - Tug | 5172 |
| Service - Other | 4799 |
| Miscellaneous Fishing | 8942 |
| Miscellaneous - Other | 1727 |
| Offshore | 5068 |
| Yacht | 2214 |

**Table S2| Sample vessel classification and statistics**

| Vessel Capacity Category | | |
| --- | --- | --- |
| Vessel type | Capacity | Count |
| Bulk Carrier | 0-9999 | 524 |
|  | 10000-34999 | 1603 |
|  | 35000-59999 | 2912 |
|  | 60000-99999 | 3305 |
|  | 100000-199999 | 1049 |
|  | 200000-+ | 538 |
| Chemical Tanker | 0-4999 | 1274 |
|  | 5000-9999 | 777 |
|  | 10000-19999 | 1595 |
|  | 20000-39999 | 1058 |
|  | 40000-+ | 2066 |
| Container | 0-999 | 72 |
|  | 1000-1999 | 75 |
|  | 2000-2999 | 82 |
|  | 3000-4999 | 197 |
|  | 5000-7999 | 205 |
|  | 8000-11999 | 209 |
|  | 12000-14499 | 49 |
|  | 14500-19999 | 213 |
|  | 20000-+ | 1074 |
| General cargo | 0-4999 | 2777 |
|  | 5000-9999 | 838 |
|  | 10000-19999 | 4058 |
|  | 20000-+ | 1359 |
| Liquefied gas tanker | 0-49999 | 4816 |
|  | 50000-99999 | 2964 |
|  | 100000-199999 | 132 |
|  | 200000-+ | 0 |
| Oil tanker | 0-4999 | 1394 |
|  | 5000-9999 | 70 |
|  | 10000-19999 | 50 |
|  | 20000-59999 | 364 |
|  | 60000-79999 | 684 |
|  | 80000-119999 | 2761 |
|  | 120000-199999 | 1621 |
|  | 200000-+ | 2337 |
| Others | 0-+ | 34511 |
| Engine Tier Category | | |
| Tier 0 (engine construction date before 2000) | | 29058 |
| Tier 1 (engine construction date between 2000-2010) | | 21614 |
| Tier 2 (engine construction date between 2011-2015) | | 15695 |
| Tier 3(engine construction date after 2016) | | 13246 |

**Table S3| AIS message broadcast frequency**

| Transponder Type | Vessel's Moving Status (Transponder ON) | Transmission Rate |
| --- | --- | --- |
| Class A | Anchored / Moored | Every 3 Minutes |
| Class A | Sailing 0-14 knots | Every 10 Seconds |
| Class A | Sailing 14-23 knots | Every 6 Seconds |
| Class A | Sailing 0-14 knots and changing course | Every 3.33 Seconds |
| Class A | Sailing 14-23 knots and changing course | Every 2 Seconds |
| Class A | Sailing faster than 23 knots | Every 2 Seconds |
| Class A | Sailing faster than 23 knots and changing course | Every 2 Seconds |
| Class B | Stopped or sailing up to 2 knots | Every 3 Minutes |
| Class B | Sailing faster than 2 knots | Every 30 Seconds |

**Figure S1. Geographic-based high seas shipping allocation.** In comparison to the existing bottom-up shipping estimation approaches such as vessel-based and voyage-based shipping calculation method, we collect AIS messages for both international and domestic shipping routes and all kinds of vessel types if and only if the AIS messages are reported from high seas regions (yellow bar in Figure S1). As a result, exclusive economic zones AIS messages and shipping emission (grey bar in Figure S1) are excluded in this study.

**Figure S2. Technical strategy for Geographic-based Emission Estimation Model (GEEM)**

**Table S4| Emission factors for vessel main engine (g/kwh)**

| Engine  Type | Fuel  Type | Tier | Model Year | PM_2.5_ | PM_10_ | NO_x_ | SO_2_ | CO | NM VOC | CO_2_ | N_2_O | CH_4_ |
| --- | --- | --- | --- | --- | --- | --- | --- | --- | --- | --- | --- | --- |
| SSD | HFO  (2.43%  Sulfur) | Tier 0 | <1999 | 1.28 | 1.39 | 18.1 | 8.751 | 0.54 | 0.632 | 576 | 0.031 | 0.012 |
| MSD |  |  |  | 1.28 | 1.39 | 14.0 | 9.224 | 0.54 | 0.527 | 607 | 0.034 | 0.01 |
| HSD |  |  |  | 1.29 | 1.4 | 10.0 | 9.697 | 0.54 | 0.527 | 638 | 0.030 | 0.01 |
| SSD |  | Tier 1 | 2000-2010 | 1.28 | 1.39 | 17.0 | 8.278 | 0.54 | 0.632 | 545 | 0.031 | 0.012 |
| MSD |  |  |  | 1.28 | 1.39 | 13.0 | 8.751 | 0.54 | 0.527 | 576 | 0.034 | 0.01 |
| HSD |  |  |  | 1.28 | 1.39 | 9.8 | 9.224 | 0.54 | 0.527 | 607 | 0.030 | 0.01 |
| SSD |  | Tier 2 | 2011-2015 | 1.28 | 1.39 | 14.4 | 8.593 | 0.54 | 0.632 | 545 | 0.031 | 0.012 |
| MSD |  |  |  | 1.28 | 1.39 | 10.5 | 9.084 | 0.54 | 0.527 | 576 | 0.034 | 0.01 |
| HSD |  |  |  | 1.28 | 1.39 | 7.7 | 9.575 | 0.54 | 0.527 | 607 | 0.030 | 0.01 |
| SSD |  | Tier 3 | >2016 | 1.28 | 1.39 | 14.4 | 8.890 | 0.54 | 0.632 | 545 | 0.031 | 0.01 |
| MSD |  |  |  | 1.28 | 1.39 | 10.5 | 9.398 | 0.54 | 0.527 | 576 | 0.034 | 0.01 |
| HSD |  |  |  | 1.28 | 1.39 | 7.7 | 9.906 | 0.54 | 0.527 | 607 | 0.030 | 0.01 |
| SSD | MDO/  MGO  (0.13%  Sulfur) | Tier 0 | <1999 | 0.17 | 0.18 | 18.1 | 0.508 | 0.044 | 0.632 | 561 | 0.030 | 0.012 |
| MSD |  |  |  | 0.17 | 0.18 | 14.0 | 0.537 | 0.046 | 0.527 | 593 | 0.030 | 0.01 |
| HSD |  |  |  | 0.17 | 0.18 | 10.0 | 0.551 | 0.54 | 0.527 | 609 | 0.034 | 0.01 |
| SSD |  | Tier 1 | 2000-2010 | 0.17 | 0.19 | 17.0 | 0.479 | 0.044 | 0.632 | 529 | 0.030 | 0.012 |
| MSD |  |  |  | 0.17 | 0.18 | 13.0 | 0.508 | 0.046 | 0.527 | 561 | 0.030 | 0.01 |
| HSD |  |  |  | 0.17 | 0.18 | 9.8 | 0.537 | 0.54 | 0.527 | 593 | 0.034 | 0.01 |
| SSD |  | Tier 2 | 2011-2015 | 0.17 | 0.19 | 14.4 | 0.446 | 0.044 | 0.632 | 529 | 0.030 | 0.012 |
| MSD |  |  |  | 0.17 | 0.18 | 10.5 | 0.473 | 0.046 | 0.527 | 561 | 0.030 | 0.01 |
| HSD |  |  |  | 0.17 | 0.18 | 7.7 | 0.500 | 0.54 | 0.527 | 593 | 0.034 | 0.01 |
| SSD |  | Tier 3 | >2016 | 0.17 | 0.19 | 14.4 | 0.231 | 0.044 | 0.632 | 529 | 0.030 | 0.01 |
| MSD |  |  |  | 0.17 | 0.18 | 10.5 | 0.245 | 0.046 | 0.527 | 561 | 0.030 | 0.01 |
| HSD |  |  |  | 0.17 | 0.18 | 7.7 | 0.259 | 0.54 | 0.527 | 593 | 0.034 | 0.01 |
| Otto | LNG | Tier 0-2 | <2016 | 0.03 | 0.03 | 1.3 | 0.003 | 1.3 | 0.5 | 457 | 0.018 | 8.5 |
| Otto (MS) | LNG | Tier 3 | >2016 | 0.02 | 0.02 | 1.3 | 0.003 | 1.3 | 0.500 | 457 | 0.02 | 5.5 |
| Otto (SS) |  |  |  | 0.02 | 0.02 | 1.3 | 0.003 | 1.3 | 0.500 | 457 | 0.02 | 2.5 |
| Diesel |  |  |  | 0.01 | 0.01 | 3.4 | 0.003 | 1.04 | 0.400 | 457 | 0.03 | 0.2 |
| Note: 1) SSD: Slow Speed Diesel; MSD: Medium Speed Diesel; HSD: High Speed Diesel; HFO: Heavy Fuel Oil; MDO: Marine Diesel Oil; MGO: Marine Gas Oil; LNG: Liquefied Natural Gas; Otto: Otto-cycle LNG-fueled engine.  2) According to the Fourth IMO GHG Study, we set Heavy Fuel Oil (HFO) average sulfur content at 2.43%, Marine Diesel Oil (MDO) and Marine Gas Oil (MGO) at 0.13%. In addition, we collect and update all types of emission factors (PM_2.5,_ PM_10,_ NO_x,_ SO_2,_ CO, NMVOC, CO_2,_ N_2_O, CH_4_) reported in Fourth IMO GHG Study. | | | | | | | | | | | | |

**Table S5| Adjusted emission factors for vessel main engines at low loads (%)**

| Load Factor (%) | PM | NO_x_ | SO_2_ | CO | NMVOC | CO_2_ | N_2_O | CH_4_ |
| --- | --- | --- | --- | --- | --- | --- | --- | --- |
| 2 | 7.29 | 4.63 | 1 | 9.7 | 21.18 | 1 | 4.63 | 21.18 |
| 3 | 4.33 | 2.92 | 1 | 6.49 | 11.68 | 1 | 2.92 | 11.68 |
| 4 | 3.09 | 2.21 | 1 | 4.86 | 7.71 | 1 | 2.21 | 7.71 |
| 5 | 2.44 | 1.83 | 1 | 3.9 | 5.61 | 1 | 1.83 | 5.61 |
| 6 | 2.04 | 1.6 | 1 | 3.26 | 4.35 | 1 | 1.6 | 4.35 |
| 7 | 1.79 | 1.45 | 1 | 2.8 | 3.52 | 1 | 1.45 | 3.52 |
| 8 | 1.61 | 1.35 | 1 | 2.45 | 2.95 | 1 | 1.35 | 2.95 |
| 9 | 1.48 | 1.27 | 1 | 2.18 | 2.52 | 1 | 1.27 | 2.52 |
| 10 | 1.38 | 1.22 | 1 | 1.97 | 2.18 | 1 | 1.22 | 2.18 |
| 11 | 1.3 | 1.17 | 1 | 1.79 | 1.96 | 1 | 1.17 | 1.96 |
| 12 | 1.24 | 1.14 | 1 | 1.64 | 1.76 | 1 | 1.14 | 1.76 |
| 13 | 1.19 | 1.11 | 1 | 1.52 | 1.6 | 1 | 1.11 | 1.6 |
| 14 | 1.15 | 1.08 | 1 | 1.41 | 1.47 | 1 | 1.08 | 1.47 |
| 15 | 1.11 | 1.06 | 1 | 1.32 | 1.36 | 1 | 1.06 | 1.36 |
| 16 | 1.08 | 1.05 | 1 | 1.24 | 1.26 | 1 | 1.05 | 1.26 |
| 17 | 1.06 | 1.03 | 1 | 1.17 | 1.18 | 1 | 1.03 | 1.18 |
| 18 | 1.04 | 1.02 | 1 | 1.11 | 1.11 | 1 | 1.02 | 1.11 |
| 19 | 1.02 | 1.01 | 1 | 1.05 | 1.05 | 1 | 1.01 | 1.05 |
| 20 | 1 | 1 | 1 | 1 | 1 | 1 | 1 | 1 |

**Table S6| Emission factors for auxiliary engine (g/kwh)**

| Engine  Type | Fuel  Type | Tier | Model Year | PM_2.5_ | PM_10_ | NO_x_ | SO_x_ | CO | NM VOC | CO_2_ | N_2_O | CH_4_ |
| --- | --- | --- | --- | --- | --- | --- | --- | --- | --- | --- | --- | --- |
| SSD | HFO  (2.43%  Sulfur) | Tier 0 | <1999 | 1.29 | 1.4 | 11.2 | 9.697 | 0.54 | 0.421 | 638 | 0.040 | 0.01 |
| MSD |  |  |  | 1.29 | 1.4 | 11.2 | 9.697 | 0.54 | 0.421 | 638 | 0.040 | 0.01 |
| HSD |  |  |  | 1.29 | 1.4 | 11.2 | 9.697 | 0.54 | 0.421 | 638 | 0.040 | 0.01 |
| SSD |  | Tier 1 | 2000-2010 | 1.28 | 1.39 | 11.2 | 9.224 | 0.54 | 0.421 | 607 | 0.040 | 0.01 |
| MSD |  |  |  | 1.28 | 1.39 | 11.2 | 9.224 | 0.54 | 0.421 | 607 | 0.040 | 0.01 |
| HSD |  |  |  | 1.28 | 1.39 | 11.2 | 9.224 | 0.54 | 0.421 | 607 | 0.040 | 0.01 |
| SSD |  | Tier 2 | 2011-2015 | 1.28 | 1.39 | 11.2 | 9.575 | 0.54 | 0.421 | 607 | 0.040 | 0.01 |
| MSD |  |  |  | 1.28 | 1.39 | 11.2 | 9.575 | 0.54 | 0.421 | 607 | 0.040 | 0.01 |
| HSD |  |  |  | 1.28 | 1.39 | 11.2 | 9.575 | 0.54 | 0.421 | 607 | 0.040 | 0.01 |
| SSD |  | Tier 3 | >2016 | 1.28 | 1.39 | 11.2 | 9.906 | 0.54 | 0.421 | 607 | 0.040 | 0.01 |
| MSD |  |  |  | 1.28 | 1.39 | 11.2 | 9.906 | 0.54 | 0.421 | 607 | 0.040 | 0.01 |
| HSD |  |  |  | 1.28 | 1.39 | 11.2 | 9.906 | 0.54 | 0.421 | 607 | 0.040 | 0.01 |
| SSD | MDO/  MGO  (0.13%  Sulfur) | Tier 0 | <1999 | 0.17 | 0.18 | 11.2 | 0.551 | 0.54 | 0.421 | 609 | 0.036 | 0.01 |
| MSD |  |  |  | 0.17 | 0.18 | 11.2 | 0.551 | 0.54 | 0.421 | 609 | 0.036 | 0.01 |
| HSD |  |  |  | 0.17 | 0.18 | 11.2 | 0.551 | 0.54 | 0.421 | 609 | 0.036 | 0.01 |
| SSD |  | Tier 1 | 2000-2010 | 0.17 | 0.18 | 11.2 | 0.537 | 0.54 | 0.421 | 593 | 0.036 | 0.01 |
| MSD |  |  |  | 0.17 | 0.18 | 11.2 | 0.537 | 0.54 | 0.421 | 593 | 0.036 | 0.01 |
| HSD |  |  |  | 0.17 | 0.18 | 11.2 | 0.537 | 0.54 | 0.421 | 593 | 0.036 | 0.01 |
| SSD |  | Tier 2 | 2011-2015 | 0.17 | 0.18 | 11.2 | 0.500 | 0.54 | 0.421 | 593 | 0.036 | 0.01 |
| MSD |  |  |  | 0.17 | 0.18 | 11.2 | 0.500 | 0.54 | 0.421 | 593 | 0.036 | 0.01 |
| HSD |  |  |  | 0.17 | 0.18 | 11.2 | 0.500 | 0.54 | 0.421 | 593 | 0.036 | 0.01 |
| SSD |  | Tier 3 | >2016 | 0.17 | 0.18 | 11.2 | 0.259 | 0.54 | 0.421 | 593 | 0.036 | 0.01 |
| MSD |  |  |  | 0.17 | 0.18 | 11.2 | 0.259 | 0.54 | 0.421 | 593 | 0.036 | 0.01 |
| HSD |  |  |  | 0.17 | 0.18 | 11.2 | 0.259 | 0.54 | 0.421 | 593 | 0.036 | 0.01 |
| Otto | LNG | Na | all | 0.02 | 0.02 | 1.3 | 0.005 | 1.3 | 0.5 | 429 | 0.018 | 5.5 |
| Note: The emission factor of engine tier 3 NOx can only be used within the North Sea and Baltic Sea NOx Emission Control Area (NECA). We substitute the engine tier 3 NOx emission factor with engine tier 2 NOx emission in other high seas. | | | | | | | | | | | | |

**Table S7|** **Adjusted auxiliary engine and boiler power output (kw)**

| Ship Type | Size | Auxiliary Boiler Power Output | | | | Auxiliary Engine Power Output | | | |
| --- | --- | --- | --- | --- | --- | --- | --- | --- | --- |
|  |  | At berth | Anchored | Maneuvering | Sea | At berth | Anchored | Maneuvering | Sea |
| Bulk carrier | 0-9,999 | 70 | 70 | 60 | 0 | 110 | 180 | 500 | 190 |
|  | 10,000-34,999 | 70 | 70 | 60 | 0 | 110 | 180 | 500 | 190 |
|  | 35,000-59,999 | 130 | 130 | 120 | 0 | 150 | 250 | 680 | 260 |
|  | 60,000-99,999 | 260 | 260 | 240 | 0 | 240 | 400 | 1,100 | 410 |
|  | 100,000-199,999 | 260 | 260 | 240 | 0 | 240 | 400 | 1,100 | 410 |
|  | 200,000-+ | 260 | 260 | 240 | 0 | 240 | 400 | 1,100 | 410 |
| Chemical  tanker | 0-4,999 | 670 | 160 | 130 | 0 | 110 | 170 | 190 | 200 |
|  | 5,000-9,999 | 670 | 160 | 130 | 0 | 330 | 490 | 560 | 580 |
|  | 10,000-19,999 | 1,000 | 240 | 200 | 0 | 330 | 490 | 560 | 580 |
|  | 20,000-39,999 | 1,350 | 320 | 270 | 0 | 790 | 550 | 900 | 660 |
|  | 40,000-+ | 1,350 | 320 | 270 | 0 | 790 | 550 | 900 | 660 |
| Container | 0-999 | 250 | 250 | 240 | 0 | 370 | 450 | 790 | 410 |
|  | 1,000-1,999 | 340 | 340 | 310 | 0 | 820 | 910 | 1,750 | 900 |
|  | 2,000-2,999 | 460 | 450 | 430 | 0 | 610 | 910 | 1,900 | 920 |
|  | 3,000-4,999 | 480 | 480 | 430 | 0 | 1,100 | 1,350 | 2,500 | 1,400 |
|  | 5,000-7,999 | 590 | 580 | 550 | 0 | 1,100 | 1,400 | 2,800 | 1,450 |
|  | 8,000-11,999 | 620 | 620 | 540 | 0 | 1,150 | 1,600 | 2,900 | 1,800 |
|  | 12,000-14,499 | 630 | 630 | 630 | 0 | 1,300 | 1,800 | 3,250 | 2,050 |
|  | 14,500-19,999 | 630 | 630 | 630 | 0 | 1,400 | 1,950 | 3,600 | 2,300 |
|  | 20,000-+ | 700 | 700 | 700 | 0 | 1,400 | 1,950 | 3,600 | 2,300 |
| General cargo | 0-4,999 | 0 | 0 | 0 | 0 | 90 | 50 | 180 | 60 |
|  | 5,000-9,999 | 110 | 110 | 100 | 0 | 240 | 130 | 490 | 180 |
|  | 10,000-19,999 | 150 | 150 | 130 | 0 | 720 | 370 | 1,450 | 520 |
|  | 10,000-+ | 150 | 150 | 130 | 0 | 720 | 370 | 1,450 | 520 |
| Liquefied gas tanker | 0-49,999 | 1,000 | 200 | 200 | 100 | 240 | 240 | 360 | 240 |
|  | 50,000-99,999 | 1,000 | 200 | 200 | 100 | 1,700 | 1,700 | 2,600 | 1,700 |
|  | 100,000-199,999 | 1,500 | 300 | 300 | 150 | 2,500 | 2,000 | 2,300 | 2,650 |
|  | 200,000-+ | 3,000 | 600 | 600 | 300 | 6,750 | 7,200 | 7,200 | 6,750 |
| Oil tanker | 0-4,999 | 500 | 100 | 100 | 0 | 250 | 250 | 375 | 250 |
|  | 5,000-9,999 | 750 | 150 | 150 | 0 | 375 | 375 | 560 | 375 |
|  | 10,000-19,999 | 1,250 | 250 | 250 | 0 | 690 | 500 | 580 | 490 |
|  | 20,000-59,999 | 2,700 | 270 | 270 | 270 | 720 | 520 | 600 | 510 |
|  | 60,000-79,999 | 3,250 | 360 | 360 | 280 | 620 | 490 | 770 | 560 |
|  | 80,000-119,999 | 4,000 | 400 | 400 | 280 | 800 | 640 | 910 | 690 |
|  | 120,000-199,999 | 6,500 | 500 | 500 | 300 | 2,500 | 770 | 1,300 | 860 |
|  | 200,000-+ | 7,000 | 600 | 600 | 300 | 2,500 | 770 | 1,300 | 860 |
| Fishing | 0-+ | 0 | 0 | 0 | 0 | 200 | 200 | 200 | 200 |
| Note: Vessel movement modes can be categorized into 4 types by their speed and maximum continuous rated power (MCR). Specifically, At berth = vessel speed less than 1 knot, At anchorage = vessel speed between 1 knot-3 knot, Maneuvering = vessel speed great than 3 knots and less than 20% MCR, At sea = vessel speed above 20% MCR. Vessels with different movement modes and types have heterogenous boiler power output, auxiliary engine power output and GHG emission patterns. | | | | | | | | | |

**Table S8| Emission factor for boiler (g/kwh)**

| Fuel type | PM_2.5_ | PM_10_ | NO_x_ | SO_2_ | CO | NMVOC | CO_2_ | N_2_O | CH_4_ |
| --- | --- | --- | --- | --- | --- | --- | --- | --- | --- |
| HFO (2.43%Sulfur) | 1.31 | 1.42 | 2.1 | 14.85 | 0.2 | 0.105 | 1059 | 0.040 | 0.002 |
| MDO/MGO (0.13%Sulfur) | 0.13 | 0.14 | 2.1 | 0.825 | 0.2 | 0.105 | 970 | 0.049 | 0.002 |
| LNG | 0.03 | 0.03 | 1.3 | 0.00269 | 0.2 | 0.105 | 457 | 0.020 | 0.04 |

**Table S9| Static dataset comparison**

| Ship Type | IMO Dataset | This Study | % of IMO Dataset |
| --- | --- | --- | --- |
| Bulk Carrier | 11672 | 11981 | 96.5% |
| Oil Tanker | 8177 | 7403 | 90.5% |
| Container | 5182 | 5366 | 103.6% |
| General Cargo | 14994 | 13196 | 88.0% |
| Chemical Tanker | 5506 | 5145 | 93.4% |
| Ro-Ro | 2002 | 1162 | 58.0% |
| Cruise | 612 | 475 | 77.6% |
| Refrigerated Bulk | 895 | 684 | 76.4% |
| Liquefied Gas Tanker | 1953 | 2011 | 102.9% |
| Other Liquids Tankers | 179 | 34 | 18.9% |
| Ferry - Pax Only | 3459 | 1272 | 36.77% |
| Ferry - Ro-Pax | 3148 | 2120 | 67.3% |
| Vehicle | 828 | 842 | 101.7% |
| Service - Tug | 20251 | 5172 | 25.5% |
| Service - Other | 6180 | 4799 | 77.6% |
| Miscellaneous Fishing | 23911 | 8942 | 37.4% |
| Miscellaneous - Other | 645 | 1727 | 267.7% |
| Offshore | 7555 | 5068 | 67.8% |
| Yacht | 2477 | 2214 | 89.4% |

**Table S10| AIS dataset coverage**

| Ship Type | 2015 | 2016 | 2017 | 2018 | 2019 | Ships in Service | % if In-service Ships Observed on AIS |
| --- | --- | --- | --- | --- | --- | --- | --- |
| Bulk Carrier | 10544 | 10877 | 11286 | 11258 | 11981 | 12327 | 97.2% |
| Oil Tanker | 5807 | 5940 | 6293 | 6403 | 7403 | 8044 | 92.0% |
| Container | 5100 | 5187 | 5377 | 5328 | 5366 | 5367 | 99.9% |
| General Cargo | 12166 | 12282 | 12463 | 13196 | 13196 | 13940 | 94.7% |
| Chemical Tanker | 4400 | 4593 | 4785 | 4837 | 5145 | 5843 | 88.1% |
| RoRo | 1033 | 1055 | 1110 | 1098 | 1162 | 1631 | 71.2% |
| Cruise | 399 | 404 | 416 | 417 | 475 | 481 | 98.8% |
| Refrigerated Bulk | 686 | 670 | 677 | 651 | 684 | 792 | 86.4% |
| Liquefied Gas Tanker | 1696 | 1802 | 1912 | 1909 | 2011 | 2136 | 94.1% |
| Other Liquid Tankers | 33 | 33 | 33 | 32 | 34 | 87 | 39.1% |
| Ferry-PaxOnly | 1161 | 1181 | 1206 | 1202 | 1272 | 1400 | 90.8% |
| Ferry-RoPax | 1815 | 1879 | 1950 | 1946 | 2120 | 2798 | 75.8% |
| Vehicle | 774 | 793 | 825 | 784 | 842 | 873 | 96.4% |
| Service Tug | 4620 | 4752 | 4903 | 4834 | 5172 | 5569 | 92.9% |
| Service Other | 4647 | 4642 | 4709 | 4642 | 4799 | 5267 | 91.1% |
| Miscellaneous Fishing | 8393 | 8547 | 8682 | 8632 | 8942 | 9761 | 91.6% |
| Miscellaneous Other | 1541 | 1569 | 1637 | 1599 | 1727 | 2553 | 67.6% |
| Offshore | 5157 | 5107 | 4998 | 4838 | 5068 | 5638 | 89.9% |

**Table S11| the time interval statistics for AIS data**

| Time Internal | Percentage |
| --- | --- |
| 30 seconds | 92.28% |
| 60 seconds | 3.33% |
| 90 seconds | 0.85% |
| 120 seconds | 0.31% |
| 20 minutes | 2.99% |
| 30 minutes | 0.19% |
| > 30 minutes | 0.05% |

**Table S12| updated emission factors for PM10 (g/kwh)**

| Generation | Engine | Fuel | 2015 | 2016 | 2017 | 2018 | 2019 |
| --- | --- | --- | --- | --- | --- | --- | --- |
| 1 | SSD | HFO | 1.35 | 1.39 | 1.4 | 1.4 | 1.4 |
|  |  | MDO | 0.18 | 0.18 | 0.18 | 0.18 | 0.18 |
|  | MSD | HFO | 1.35 | 1.39 | 1.4 | 1.4 | 1.4 |
|  |  | MDO | 0.18 | 0.18 | 0.18 | 0.18 | 0.18 |
|  | HSD | HFO | 1.35 | 1.39 | 1.4 | 1.4 | 1.4 |
|  |  | MDO | 0.18 | 0.18 | 0.18 | 0.17 | 0.17 |
|  | Boiler | HFO | 1.34 | 1.41 | 1.42 | 1.42 | 1.42 |
|  |  | MDO | 0.15 | 0.15 | 0.15 | 0.14 | 0.14 |
|  |  | LNG | 0.03 | 0.03 | 0.03 | 0.03 | 0.03 |
|  | AE | HFO | 1.35 | 1.39 | 1.4 | 1.4 | 1.4 |
|  |  | MDO | 0.18 | 0.18 | 0.18 | 0.17 | 0.17 |
| 2 | SSD | HFO | 1.35 | 1.38 | 1.39 | 1.39 | 1.39 |
|  |  | MDO | 0.19 | 0.19 | 0.19 | 0.18 | 0.18 |
|  | MSD | HFO | 1.35 | 1.39 | 1.39 | 1.39 | 1.39 |
|  |  | MDO | 0.18 | 0.18 | 0.18 | 0.18 | 0.18 |
|  | HSD | HFO | 1.35 | 1.39 | 1.4 | 1.4 | 1.4 |
|  |  | MDO | 0.18 | 0.18 | 0.18 | 0.18 | 0.18 |
|  | Otto-MS | LNG | 0.02 | 0.02 | 0.02 | 0.02 | 0.02 |
|  | Boiler | HFO | 1.34 | 1.41 | 1.42 | 1.42 | 1.42 |
|  |  | MDO | 0.15 | 0.15 | 0.15 | 0.14 | 0.14 |
|  |  | LNG | 0.03 | 0.03 | 0.03 | 0.03 | 0.03 |
|  | AE | HFO | 1.35 | 1.39 | 1.4 | 1.4 | 1.4 |
|  |  | MDO | 0.18 | 0.18 | 0.18 | 0.18 | 0.18 |
|  |  | LNG | 0.02 | 0.02 | 0.02 | 0.02 | 0.02 |
| 3 | SSD | HFO | 1.36 | 1.34 | 1.35 | 1.35 | 1.35 |
|  |  | MDO | 0.2 | 0.2 | 0.2 | 0.19 | 0.19 |
|  | MSD | HFO | 1.36 | 1.34 | 1.35 | 1.35 | 1.35 |
|  |  | MDO | 0.2 | 0.2 | 0.2 | 0.19 | 0.19 |
|  | HSD | HFO | 1.37 | 1.34 | 1.35 | 1.35 | 1.35 |
|  |  | MDO | 0.2 | 0.2 | 0.2 | 0.18 | 0.18 |
|  | Otto-SS | LNG | 0.02 | 0.02 | 0.02 | 0.02 | 0.02 |
|  | Otto-MS | LNG | 0.02 | 0.02 | 0.02 | 0.02 | 0.02 |
|  | LNG-Diesel | LNG | 0.01 | 0.01 | 0.01 | 0.01 | 0.01 |
|  | Boiler | HFO | 1.38 | 1.33 | 1.35 | 1.34 | 1.34 |
|  |  | MDO | 0.18 | 0.17 | 0.17 | 0.15 | 0.15 |
|  |  | LNG | 0.03 | 0.03 | 0.03 | 0.03 | 0.03 |
|  | AE | HFO | 1.37 | 1.34 | 1.35 | 1.35 | 1.35 |
|  |  | MDO | 0.2 | 0.2 | 0.2 | 0.18 | 0.18 |
|  |  | LNG | 0.02 | 0.02 | 0.02 | 0.02 | 0.02 |
| Note: SSD: Slow Speed Diesel; MSD: Medium Speed Diesel; HSD: High Speed Diesel; HFO: Heavy Fuel Oil; MDO: Marine Diesel Oil; MGO: Marine Gas Oil; LNG: Liquefied Natural Gas; Otto: Otto-cycle LNG-fueled engine. Generations 1 to 3 indicate engine built before 1984, between 1984 and 2000 and after 2000 respectively. | | | | | | | |

**Table S13| updated emission factors for SO2 (g/kwh)**

| Engine Type | Fuel Type | 2015 | 2016 | 2017 | 2018 | 2019 |
| --- | --- | --- | --- | --- | --- | --- |
| SSD | HFO | 8.383 | 8.820 | 8.890 | 8.890 | 8.890 |
|  | MDO | 0.264 | 0.264 | 0.264 | 0.231 | 0.231 |
| MSD | HFO | 8.862 | 9.324 | 9.398 | 9.398 | 9.398 |
|  | MDO | 0.280 | 0.280 | 0.280 | 0.245 | 0.245 |
| HSD | HFO | 9.341 | 9.828 | 9.906 | 9.906 | 9.906 |
|  | MDO | 0.296 | 0.296 | 0.296 | 0.259 | 0.259 |
| LNG-Otto (MS) | LNG | 0.005 | 0.005 | 0.005 | 0.005 | 0.005 |
| LNG-Otto (SS) | LNG | 0.006 | 0.006 | 0.006 | 0.006 | 0.006 |
| LNG-Diesel | LNG | 0.014 | 0.014 | 0.014 | 0.013 | 0.013 |
| Boiler | HFO | 16.286 | 17.136 | 17.272 | 17.272 | 17.272 |
|  | MDO | 0.512 | 0.512 | 0.512 | 0.448 | 0.448 |
|  | LNG | 0.009 | 0.009 | 0.009 | 0.009 | 0.009 |
| Auxiliary Engines | HFO | 9.341 | 9.828 | 9.906 | 9.906 | 9.906 |
|  | MDO | 0.296 | 0.296 | 0.296 | 0.259 | 0.259 |
|  | LNG | 0.005 | 0.005 | 0.005 | 0.005 | 0.005 |

1. * Corresponding authors’ e-mails: sywang@amss.ac.cn; guandabo@tsinghua.edu.cn [↑](#footnote-ref-1)
